# Development of Communication Skills Training for Oncology Clinicians to Promote Inclusion of the Family Members of LGBTQ+ People with Cancer

**DOI:** 10.3390/healthcare12242557

**Published:** 2024-12-19

**Authors:** William E. Rosa, Amanda Kastrinos, Smita C. Banerjee, Kimberly Acquaviva, Koshy Alexander, Meghan McDarby, Mia Behrens, Patricia A. Parker

**Affiliations:** 1 Department of Psychiatry and Behavioral Sciences, Memorial Sloan Kettering Cancer Center, New York, NY 10017, USA; 2 University of Virginia School of Nursing, Charlottesville, VA 22903, USA; 3 Department of Medicine (Geriatrics), Memorial Sloan Kettering Cancer Center, New York, NY 10065, USA

**Keywords:** cancer communication, chosen family, decision-making, empathic communication, health education, health equity, LGBT, LGBTQ+, SGM, sexual and gender minorities, transgender

## Abstract

**Background:** LGBTQ+ persons with cancer and their families consistently face discriminatory care. In addition, clinicians report inadequate population-specific knowledge and communication skills to effectively promote LGBTQ+ inclusion. To fill this gap, we designed a communication skills training based on extant literature; multidisciplinary perspectives; and patient, family, and clinician expert engagement. **Methods:** Training content comprised didactic information, including exemplar videos of communication strategies, and experiential learning roleplay opportunities with standardized patient and family member actors. Two training sessions were conducted virtually with invited multidisciplinary clinicians using convenience sampling. Each training was followed by a one-hour, semi-structured focus group to solicit feedback on participants’ experiences and recommendations for improvement. Focus group transcripts were thematically analyzed using a constant comparative approach. **Results:** Two major themes were identified: key takeaways from the training and recommendations for improvement. Participants reported favorable learning experiences and believed the training would have a positive influence on future clinical interactions. We synthesized recommendations into five discrete pieces of feedback related to (1) the advanced level of training material; (2) diversity throughout the training; (3) complexity of family dynamics; and (4) recovery from communication mistakes; and (5) additional topics for future training. Based on feedback, corresponding changes for each item and a refined communication skills blueprint are provided. **Conclusions:** Our innovative training on inclusive cancer care for LGBTQ+ patients’ families will provide both knowledge-based and experiential learning opportunities to advance clinicians’ confidence in communicating empathically with members of diverse sexual orientation and gender identities. The training is poised for feasibility and efficacy testing.

## 1. Introduction

LGBTQ+ (e.g., lesbian, gay, bisexual, transgender, queer and/or questioning) people worldwide face interpersonal, institutional, systemic, and structural inequities associated with detrimental health and social outcomes, including (but not limited to) increased risks of psychological distress, substance abuse, suicide, homelessness, and social isolation [1,2,3,4,5]. Specific to oncology, LGBTQ+ cancer survivors have an increased age-adjusted prevalence for a range of chronic diseases (e.g., heart disease, asthma, kidney disease, disability, depression) [6]. Fear or worry about mistreatment, as well as lower insurance coverage rates and lower satisfaction with cancer care, may all contribute to LGBTQ+ patients choosing to delay seeking medical attention, stopping treatment, or avoiding the health system altogether [1,7,8].

### 1.1. Discriminatory Care of LGBTQ+ Patients

In the serious illness context, LGBTQ+ patients report high levels of disrespectful and inadequate care [9,10]. Patients have reported experiencing insensitivity from clinicians due to their sexual orientation and/or gender identity (SOGI), facing a lack of awareness from clinicians regarding LGBTQ+-specific health needs, feeling judged, experiencing rudeness, and being misgendered by clinical staff (i.e., incorrect use of patient-identified pronouns) [11,12]. Clinicians confirm these reports by LGBTQ+ patients. For instance, in one study of 865 interdisciplinary palliative care and hospice clinicians, more than half thought these groups were more likely to receive discriminatory care than non-LGBTQ+ patients, with 24% and 21% having directly observed such discrimination for LGB and trans patients, respectively [13].

### 1.2. Discriminatory Care of LGBTQ+ Patients’ Family Members

LGBTQ+ patients often develop strong relationships within their communities that serve as ‘chosen family’—non-biological social networks that provide significant emotional and psychosocial support and buffering against mistreatment, abuse, and rejection, as well as abandonment that LGBTQ+ people may experience within their families of origin [1]. Thus, chosen families become integral to person- and family-centered decision-making in the LGBTQ+ serious illness context [14,15,16,17]. However, clinicians’ uncertainty regarding how to best engage partners, spouses, and other chosen family members of LGBTQ+ people may prevent inclusive communication [18,19].

The families and partners of LGBTQ+ patients have described their own discriminatory experiences: being refused access to the patient in intensive care or the emergency room, poor treatment, treatment decisions not being followed, visiting hours with the patient being limited, or being denied private time with the patient [11,20]. Such mistreatment persists throughout the disease trajectory, including in the palliative, end-of-life, and bereavement contexts [21,22,23,24]. For example, decision-making dilemmas may arise in the context of patient incapacity, an absence of advance care planning documentation, and disagreements between various chosen and biological family members [25,26]. Clinicians have reported witnessing the patient’s spouse, partner, or surrogate decision-maker being treated disrespectfully (14%) or having their decisions minimized or ignored (15%).

### 1.3. The Need for LGBTQ+ Inclusive Communication Training and Rationale for Current Study

In our previous assessments of multidisciplinary oncology clinicians’ (n = 1253) knowledge, beliefs, and communication behavior at a comprehensive cancer center, only 5% responded correctly to all seven surveyed LGBTQ+ health knowledge-based questions; about 50% answered three out of seven questions correctly [27]. Using a case-based approach to elicit information about 150 nurses’, social workers’, and chaplains’ LGBTQ+ communication knowledge and priorities in cancer care, we found that the fear of offending patients and their families, uncertainty about the legal and ethical responsibilities regarding the family of origin and chosen family decision-making, and clarifying the role of all family members were consistent challenges [25]. To improve clinicians’ skills in providing a safe and welcoming cancer care environment, an SGM (i.e., sexual and gender minority) cultural sensitivity communication training module was developed, implemented, and tested [28]. Of 33 multidisciplinary clinician participants, more than 80% responded favorably to 12 of 15 evaluation items that evaluated their engagement with and reflectiveness of experiential roleplays with LGBT standardized patients. Participants also demonstrated significant improvements in healthcare knowledge, self-efficacy, beliefs about LGBTQ+ people, and LGBTQ+-sensitive language use skills from pre- to post-training [28].

Despite advancements in LGBTQ+ inclusion in serious illness and cancer care, there is a paucity of communication skills training sessions for oncology clinicians that specifically address inclusion of the family members of LGBTQ+ patients. The current study leveraged extant literature, including our previous studies and cultural sensitivity module [11,22,25,27,28]; multidisciplinary perspectives; and patient, family, and clinician expert input to design a communication skills training module aimed at improving multidisciplinary oncology clinicians’ knowledge and self-efficacy in inclusive communication specifically for LGBTQ+ patients’ chosen and biological family members. This paper describes the module development and initial testing.

## 2. Materials and Methods

### 2.1. Study Design and Setting

**Figure 1:**
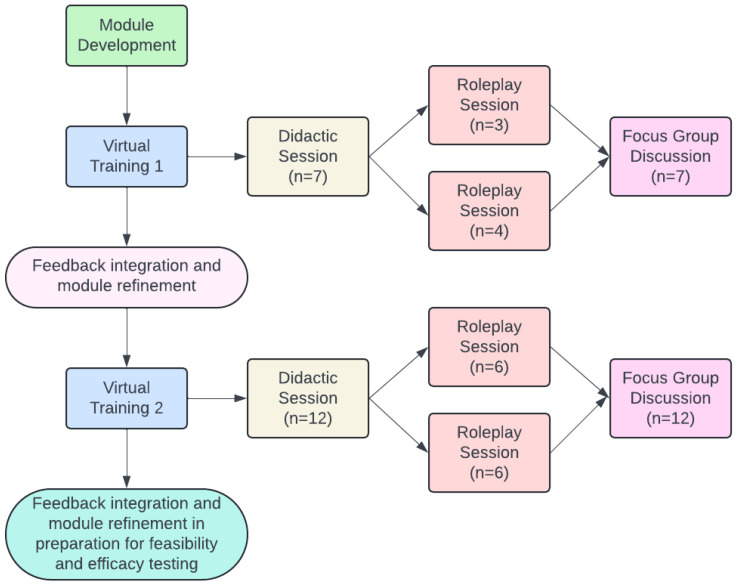
Study schema.

Module content was originally developed by a team of multidisciplinary clinicians (WER, KDA, KA, PAP) and refined through iterative meetings with Memorial Sloan Kettering Cancer Center (MSK) Communication Skills Training Program and Research Laboratory (Comskil) faculty and research trainees (SCB, MM, AK). The modules were delivered virtually through MSK Comskil using well-documented approaches to module facilitation [29,30]. Following initial development of the module, two virtual training sessions were conducted, each of which was composed of a didactic and roleplay session and followed by a focus group discussion. Feedback was integrated and the module refined following each training, as shown in the study schema (Figure 1). The study was deemed exempt by the MSK Institutional Review Board.

These initial data were collected as part of a larger study that will evaluate the module’s feasibility, acceptability, and efficacy. Participants were invited to attend one of two virtual training sessions conducted in January 2024. They attended first as module participants (i.e., learners) and then as focus group participants immediately following module delivery to discuss the overall learning experience, describe the perceived strengths and weaknesses of the module, and identify recommendations for improvement. Learning objectives for the modules include preparing participants to (1) learn strategies to create a safe and welcoming environment for LGBTQ+ patients’ family members; (2) acquire skills to explore relationships and dynamics between LGBTQ+ patients and their family members; and (3) engage LGBTQ+ patients’ family members in inclusive, respectful medical decision-making and care planning for the future.

**Figure 2:**
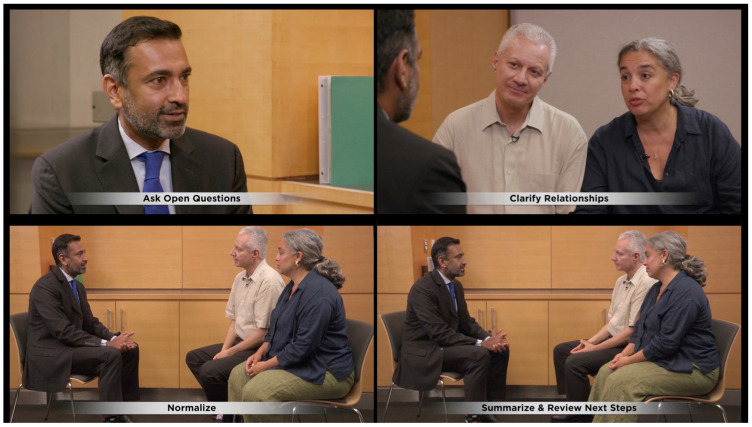
Exemplar video clips with communication skills labels. ©Memorial Sloan Kettering Cancer Center Communication Skills Training Program and Research Laboratory.

The module consisted of a 30 min didactic presentation that summarized relevant empirical literature and presented a communication blueprint comprising recommended communication strategies, skills, and process tasks. The blueprint provides all communication components of the module and guides development of all relevant teaching materials and evaluations. Our Comskil conceptual model defines *communication strategies* as a priori plans that direct communication behavior to realize a communication goal (i.e., desired outcome of a clinical encounter or consultation); *communication skills* are discrete modes (i.e., unit of speech) a clinician uses to further dialogue and achieve a strategy; and *process tasks* are sets of dialogues or nonverbal behaviors that create an environment for effective communication [29]. In addition, exemplar videos were incorporated throughout the didactic portion of the training to demonstrate the strategies, skills, and process tasks taught during the didactic. Figure 2 shows a series of clips from the exemplar video of a communication encounter between a clinician, patient, and the patient’s chosen family member. As skills are demonstrated by the clinician in the video, text overlays are provided to allow learners to easily identify them (e.g., asking open-ended questions, clarifying relationships).

Following the didactic portion of the training, participants engaged in 90 min experiential learning sessions where they roleplayed with a standardized patient and family member (i.e., professionally trained actors) to practice newly learned skills. Two cases were used in the roleplay sessions for experiential learning; these sessions were developed by an interdisciplinary team of communication and LGBTQ+ health experts. The roleplay cases used for experiential learning are provided in Table 1, and are composed of the patients’ character history, their disease and social history, and the learner’s task during the communication encounter.

### 2.2. Participants

Using a convenience sample, multidisciplinary clinicians (i.e., physicians, nurses, psychologists, advanced practice providers, clinical fellows), researchers, members of the MSK Patient & Family Advisory Council on Quality (PFACQ) and LGBTQ+ Clinical Advisory Committee, and leaders from LGBTQ+ advocacy groups participated. Diverse professions, career stages, and practice specialties were prioritized during learning pilot scheduling to promote multiple and dynamic perspectives.

### 2.3. Focus Group Procedures

At the end of each pilot training, participants completed a 1 h, semi-structured focus group soliciting feedback on their experience with the training and recommendations for improvement. The focus group guide was developed by two authors (WER & AK), who also served as moderators. Sample questions included “What could be improved to better demonstrate the need for and rationale behind this training?” and “Are there other situations or scenarios not represented in the roleplay that you think is important for clinicians to practice in creating an inclusive environment for LGBTQ+ patients and their families?” Focus groups were audio-recorded and professionally transcribed. Focus groups transcripts were thematically analyzed using the constant comparative approach [31,32]. Feedback from each focus group was discussed among Comskil faculty and incorporated to refine and improve the module following analysis (Figure 1).

## 3. Results

Seven learners from nursing (n = 2); psychology, social work, and mental health (n = 3); health communication (n = 1); and the MSK PFACQ (n = 1) attended the first training session. An additional 12 learners participated in the second training session, including nurses (n = 1); physicians (n = 6); psychology and social work (n = 2); and PFACQ representatives (n = 3). Four actors who served as standardized patients and family members during roleplays and five Comskil facilitators also participated in each focus group.

Thematic analysis of focus group transcripts identified two major themes: *key takeaways from the training* and *recommendations for improvement.* A discussion of each theme with exemplar focus group data is presented below.

### 3.1. Key Takeaways from the Training

Overall, focus group participants described positive experiences in the training session, praising its focus and the effectiveness of both the didactic presentation and roleplay session. When describing how the training makes LGBTQ+-inclusive communication skills accessible and applicable, one participant noted, “I think it’s really well done… I really appreciate how it takes things to another level” (Participant [P]1). Participants also acknowledged helpful teaching approaches that supported their learning experience between the didactic and roleplay: “One of the things I found most useful about this training in [the] didactic … was that you showed a positive example of a clinician who does it right. …Thinking back to that example was very helpful” (P2).

Several participants expressed that their participation would influence future interactions with LGBTQ+ patients and their family members. One participant said:

[You gave us] really concrete instructions around, “This is how you do introductions when there’s somebody you never met in the room before”. I’ve never been taught that. I’m 44. I’ve been doing this as an attending [physician] for 13 years and I’ve made it up. That will be the thing that will change in my practice. … Next time I have a family member in the room, I’m going to do it differently than I’ve done it before.

(P3)

Participants explained that the training also revealed the importance of using these communication skills to educate colleagues:

[I took away] the importance of not just how we communicate with patients, but how we communicate with each other amongst the health care professionals when speaking about [the] LGBTQ+ patient population… I think in our day-to-day interactions with other physicians [and] nurses… the more we role model, the better it is. And we should encourage, after just training all clinicians, to take the lead in correcting others, making sure that the correct pronouns are used even when not in front of the patient. I think those things can significantly help what will be gained by the training.

(P4)

Additional quotes related to participants’ key takeaways can be found in Table 2.

### 3.2. Recommendations for Improvement

In this theme, we synthesized participants’ recommendations into five discrete pieces of feedback, and we translated each feedback item into actionable changes to adjust the module for future testing. Each feedback item is presented below, along with its corresponding changes. Additional quotes for each feedback item can be found in Table 3.

#### 3.2.1. Feedback 1: “The Training Material Is Too Advanced”

A primary concern was that the material covered in the training may be too advanced, emphasizing that the average clinician needs support to incorporate the basics of LGBTQ+ sensitivity into their communication practices. One participant said the following:

I think some people will still be playing catch up. … We shouldn’t assume that: (a) everybody knows [the basics], and (b), that everybody who doesn’t know is because there’s… some malicious intent. And I think a lot of people just don’t know [because] they just haven’t come across [these scenarios].

(P5)

*Corresponding Training Improvements*: Participants felt that the breadth of information somewhat limited the effectiveness of the roleplays, as some participants spent more time practicing basic skills (e.g., patient and family introductions) and had less time to roleplay the more in-depth strategies and skills covered in the family inclusion training. This feedback was unsurprising, given that this training session is intended to be completed as the second in a series. In order to address this, future testing will combine the basic sensitivity training [28] prior to this more advanced content on family inclusion. This approach will allow learners to cover the basic skills and to focus on engaging both patients and families more adequately.

#### 3.2.2. Feedback 2: “There Is a Need for Greater Diversity Throughout the Training”

Participants discussed the need for greater diversity—in the broadest sense—including racial, ethnic, cultural, gender, age, and relevant topic diversity content throughout the training, as well as diversity among the actors playing patients and family caregivers. For example, one participant expressed the following:

Older populations [have] a different understanding already of this language. I was talking to a 70-year-old queer man a couple of months ago and he was talking about the word ‘queer’ … And how, for him, when he was my age, the word ‘queer’ was a derogatory term versus now for me—the word ‘queer’—that’s how I identify. That’s the word I use in my day to day. So just acknowledging the difference … [and] the growing population of young people who are identifying with these pronouns, with these terms. … [also] it is essential to have a Black trans person in this room right now. I think that’s a major voice that’s missing, in terms of [acknowledging] the mistreatment and the miscommunication. Because they’re not only dealing with their LGBTQ+ identity but their identity as a Black person or as a person of color.

(P6)

*Corresponding Training Improvements*: In direct response to participants’ feedback on the need for additional diversity, we are actively recruiting more diverse teams of facilitators and professional actors to serve as standardized patients to provide greater representation of the patients and families that participants are likely to see in practice. Our team plans to purposively sample from minoritized groups during feasibility testing to diversify the sample of learners. Iterative assessments and feedback will aid in ensuring appropriate skill-level differentiation and future module refinement to accommodate diverse and interprofessional learners with varying levels of clinical and cultural competence.

#### 3.2.3. Feedback 3: “Roleplays Scenarios Should Include More Complex Family Dynamics”

Although family histories were intentionally woven into the roleplay scenarios (see Table 1), participants welcomed the opportunity to encounter more complex family dynamics between patients and caregivers. One participant asked the following question:

How do we create… supportive language for this patient who maybe has a family member that’s there and loves them and wants to take care of them but does not sort of ascribe [to or] validate their lifestyle or their queerness? Like, how can we make sure that we’re including language that tells them—the patient—that… we’re here to support you.

(P7)

*Corresponding Training Improvements*: Family dynamics described in the current module included not only difficulties between patient and caregiver but also elements of honoring a patient’s privacy, autonomy, and choice, and inviting the challenges that patient and caregiver may be facing into clinical communication. Based on this feedback, our facilitators will intentionally be guiding our actors to increase emotional intensity and raise interpersonal tensions during roleplay to provide more complex dynamics for learners to address. Furthermore, we are currently creating additional modules that will include training sessions on high-stakes end-of-life communication and bereavement communication for LGBTQ+ patients and families that will incorporate this feedback directly into roleplay scenarios.

#### 3.2.4. Feedback 4: “We Need to Know How to Recover from Communication Mistakes”

While acknowledging the training content that informed participants how to approach clinical encounters with sensitivity and respect, it was strongly recommended that guidance on how to recover from mistakes during communication (e.g., using incorrect pronouns, assuming biological family relationships) be included. One participant made the following suggestion:

Maybe mention the kinds of things that could go wrong—the things patients and partners have told us, like bringing in biological family members at end of life who had not been involved at all in [their care previously]—maybe a few of those kinds of things to [help learners become aware]… of that type of egregious mistake. It might be helpful to think of what [it looks] like when we don’t do the right thing and to guard against that. Until you’ve had that type of experience or heard about it you may not realize just how things could go wrong.

(P8)

*Corresponding Training Improvements*: The basic sensitivity training that will be the first module provided in this series includes a focus on how to recover from mistakes and demonstrates the “dos” and “don’ts” of LGBTQ+-inclusive communication—directly addressing this feedback articulated by participants [28]. Thus, when future learners attend both training sessions in sequence, this identified priority will be met.

#### 3.2.5. Feedback 5: “Additional Topics Should Be Considered for Future Training Sessions”

Participants acknowledge that the training material would not be able to cover all potential circumstances that require empathic and inclusive communication. However, they did identify several additional topics and approaches that should be considered in future training sessions.

Maybe… the patient and the caregiver just not being on the same page… [about] an understanding of what’s happening in the first place, meaning [is the team] giving curative or palliative care?… another scenario… is just healthcare proxies. And again, a challenge there in terms of who is best equipped to be the healthcare proxy. I think situations like that obviously require… quite a bit of care and expert communication.

(P9)

*Corresponding Training Improvements*: As noted above, we are currently creating additional on high-stakes end-of-life communication and bereavement that will incorporate legal issues, such as healthcare proxies and decision-making, and other interpersonal conflicts (e.g., family member not approving of the patient’s LGBTQ+ identity).

**Table 3:** Feedback items with exemplar focus group data.

Feedback Item	Focus Group Data
Advanced level of module content	We all have different levels of comfort with having conversations about people who identify as LGBTQ+, and I’m not saying it’s right or wrong… I think we just need to be aware… That’s the truth. And there’s a lot of people who are allies who also are not sure when to [broach a certain kind of conversation] and then may just shy away because they don’t want to… offend somebody. Right? And I think somewhere in the training to acknowledge that upfront… in a very neutral way to say, “This is not about taking a stance of being woke”… but something to say, “This is really about clinical care, providing optimal, best, high quality clinical care to every patient who we see and their families”.… something that sort of sets that up so people… feel a little bit more empowered to step into [this] area. (P5) It’s imperative that clinicians have this training… I worry that we haven’t given people the basics yet… Our clinicians really have no idea how to ask [sensitive] questions and how to utilize pronouns [appropriately]. (P15) I looked at the blueprint and I thought, oh my god I have so much to learn before I go into this role play…. I found… it was a lot to learn for me in a short amount of time. (P16)
Greater diversity	If you could introduce the percentage of the older LGBTQ+ population that do not feel comfortable disclosing [their sexual orientation and gender identity]… or don’t want to share… even just a mention about that would be powerful—that not everyone, even if asked potentially, will say… they need to have that relationship built up and that culture of trust in order to share. (P13) It is imperative to have gender diversity of the actors. (P15) One of things I struggled with as someone in the community and having cancer… was with fertility and being gay with fertility. I had a doctor say [I didn’t have to worry] because there’s a second woman [in the relationship] who can carry [the pregnancy], not knowing that’s not something my wife wants to do. [Fertility might be mentioned or one of those things we use to explain] why we’re doing this [training]. (P17) The comment about intersectionality is so, so important and representation is so, so important and we just [don’t see it enough in these types of settings]. (P18)
Complexity of family dynamics	[You gave us] the background, the scenario, that’s basically telling us, “Okay, this is what’s going on”. And I wonder… [what] would happen if we had to do it on our own—how to figure it out? And [a scenario]… where there was… an unclear relationship and it wasn’t [a patient and care partner with clear male and female presentations], I also think that would be helpful”. (P12) I think it would be super helpful to have a case [where the caregiver is silencing themselves about their LGBTQ+ identity to the clinical team] as one of the teaching examples… and it is something that I feel, especially with older generations, is probably really familiar. (P19) People… don’t want to hurt the person who’s there, who is their caregiver… maybe the [clinician] should suggest at least a two minute one-on-one with just the patient because there are embarrassing things [that the patient might want to keep private]. There’s… all the medical things that you see… whether its sexual disfunction, whether it’s bathroom issues…. the dirtier side of illness. A lot of times, even in from of someone who’s important to you or a family member you don’t want to discuss. So that communication from the [clinician] with the patient about, “Let’s have two minutes by ourselves” might be helpful. (P20)
Recovery from mistakes	I wonder if it would be useful to review at the beginning to review some of the basics of diversity training like cultural humility and culturally competence and to emphasize that you’re not going to be competent [at the end of this training]… and its more about [learning how to respond] to the mistakes that you make [rather than the mistake itself]. I wonder if that’s a space to emphasize more—the after the ‘oops’ moment—and to emphasize to people that they are going to make those mistakes… also emphasizing to people that we will mistakes with any patient and how we speak to them and that we will say things that don’t align with their experience in some way, whether it’s their beliefs about treatment or about gender and sexuality… [so it’s important] to translate that whatever people learn in this module that is LGBTQ+ specific will also translate to any patient they may work with. (P2)
Additional topics to consider	I think we’re still kind of living in the ‘one partner’ world, which is kind of… heteronormative and… there’s enough people who have multiple partners… that could be added as maybe a next iteration. You’re not going to cover all the complexity in every training, but maybe again, having some acknowledgment of [that] in the training… one can imagine ‘these are the types of scenarios you may also encounter.’ (P5) Maybe an accompanying guide could [describe] certain scenarios that might come up that you [don’t have time to address during the training but could provide] suggestions or even wording… of what could be a response in a situation like this… maybe ones that aren’t the most mainstream but will come up for people, that could be very helpful… So [maybe the less common topics aren’t included in the] standardized patient scenarios, but would be helpful because they will come up and they’re not super rare either… the issues around conflict between different people involved with the patient… there might be some shadings here that would be different in an LGBTQ+ patient and partner. (P8) At some point [in the didactic] there should be a discussion of the legal paperwork—not a first discussion—[and not so much training but to raise the issue]… a lot of time if two people are together or even a larger group, the legalities get involved in terms of whose making decisions… saying ‘everyone’s in this together’ isn’t a good way to run a declining patient… I would suggest at some point there be a discussion of who is the health care proxy, have you had these types of discussions… how do you explore that the [patient and family] has thought about the legalities of the process… especially if you’re not a married couple. (P21)

### 3.3. Resulting Communication Skills Blueprint

Following integration of participant feedback, a refined communication blueprint for was finalized, as shown in Table 4. The blueprint is composed of relevant communication strategies, accompanied by corresponding communication skills and process tasks to aid clinicians in delivering inclusive and empathic communication efforts with the families of LGBTQ+ patients. Finally, the blueprint will be used in future testing of the module throughout both the didactic and experiential learning aspects of the module.

## 4. Discussion

This paper describes the development and learning pilot evaluation of a communication skills module for oncology clinicians to promote inclusive care for the families of LGBTQ+ people with cancer. The module, designed by a multidisciplinary team and refined through iterative faculty and focus group feedback, was composed of a didactic that included relevant literature and exemplar videos to demonstrate communication skills followed by an experiential roleplay session. Attendees across two virtual training sessions affirmed the importance and potential impact of the module, emphasizing their plans to integrate explicit skills into their clinical practice post-training.

Considering the favorable outcomes of the previously described LGBTQ+ basic sensitivity course [28] and the positive focus group feedback received during the current testing of the family focused module, we anticipate encouraging results as we prepare for feasibility and efficacy testing of a combined training program that will incorporate this module and the LGBTQ+ sensitivity module. This would equip participants with the basics of LGBTQ+-sensitive communication, as well as what participants in the current learning pilot identified as the more ‘advanced skills’ of empathic communication with the broader family. Testing this combined training in such a way also prepares us for large scale efficacy testing and more rigorous evaluation of post-training skill implementation across practice settings.

This training module aims to foster inclusive cancer care by equipping oncology clinicians with evidence-based communication skills applicable to their work with LGBTQ+ patients and their families. It complements other emerging health professional training sessions that seek to improve LGBTQ+ cancer health equity through a curricular focus on epidemiology, clinical research, behavioral science and interventions, and community-based participatory approaches [33,34,35]. Although our content and design was informed by an in-depth synthesis of available evidence, multidisciplinary perspectives throughout the design process, and iterative focus group input collected during the current testing, there is an opportunity to more closely partner with LGBTQ+ people facing cancer and their families throughout the study’s conduct to ensure the community’s needs, experiences, and concerns are at the fore of module content and delivery moving forward.

This study has limitations to consider. First, this was a small-scale learning pilot conducted using a convenience sample and limited to two training sessions with subsequent focus groups, lending itself to selection bias related to the participants and focus group participants. However, the invited participants represented multiple disciplines, institutions, levels of career development and corresponding clinical expertise, roles as organizational and departmental leaders, as well as patients/caregivers who are LGBTQ+ or family members of LGBTQ+ patients with cancer. Thus, perspectives sought were intentionally broad and diverse. Second, we did not include quantitative measures of participant self-efficacy; LGBTQ+-specific knowledge, beliefs, and attitudes; or module evaluation. These additional measures will be included in feasibility testing with a larger sample of participants to adequately determine the effect size. Finally, the heterogeneity of both participants and module facilitators as predominantly white prevents us from integrating the crucial insights of other individuals from historically minoritized racial, ethnic, and cultural identities. We are taking a series of steps to ensure diversity and inclusion as described in the results (see feedback item 2).

Future research will focus on feasibility testing of the LGBTQ+ family Comskil module for multidisciplinary clinicians working within our institution prior to scaling to a national learner sample. Feasibility outcomes will be based on the training recruitment rate and post-module evaluation completion rate. Acceptability outcomes will be determined through an acceptability questionnaire composed of both Likert scale and open-ended items to determine learners’ perceptions of the module’s value and helpfulness. Finally, preliminary efficacy will be assessed through pre/post measures of self-efficacy (i.e., confidence communicating with LGBTQ+ patients’ family members) and LGBTQ+ specific knowledge, beliefs, and attitudes, as well as a post-module evaluation to identify further strengths and weaknesses of all module components (i.e., the didactic lecture and experiential roleplay). The recommendations elicited from participants on important topics for future training sessions, such as legal and healthcare proxy issues, more diverse family structures, and acknowledgment of less common but still important potential scenarios, will be incorporated into additional module development on LGBTQ+ inclusive end-of-life care and bereavement communication. Future measures will include evaluation of corresponding patient and family outcomes (e.g., perceived clinician empathy) to determine learner application of communication skills post-training in the clinical setting. Thus, a long-term goal is to provide sustainable, evidence-based, and multi-module national-level training that addresses transitions across the cancer continuum, equipping multidisciplinary clinicians with the communication literacy and competencies they need to provide respectful, culturally responsive, and person- and family-centered care for LGBTQ+ people.

## 5. Conclusions

Ample evidence demonstrates that LGBTQ+ people and their families experience discriminatory care in the oncology and serious illness contexts. Despite advancements in health equity research, increased bias training requirements for clinicians, and policies that are intended to protect the rights of LGBTQ+ people, there are limited education opportunities that specifically aim to bolster the communication skills of multidisciplinary oncology clinicians and improve inclusivity for the family members of LGBTQ+ people. Building on a longstanding body of work in our communications skills training program, our innovative *Inclusive Cancer Care for LGBTQ+ Patients’ Families* module will provide both knowledge-based and experiential learning opportunities for clinicians to advance their confidence in communicating empathically with members of diverse sexual orientation and gender identities. Our early-stage testing of this module provides encouraging focus group data to prepare for future feasibility and preliminary efficacy testing and ultimately scale our LGBTQ+-inclusive communication curriculum throughout the institution and nationally.

## Figures and Tables

**Table 1:** Cases for roleplay with standardized patients and family members.

**Case 1**
Character History. Terry Davidson (she/her) works as a copyright attorney and is a partner at her law firm. She is a born-and-raised New Yorker and lives in midtown Manhattan. She has an adopted 14-year-old daughter, Sammy (she/her). For the past year, Terry has been accompanied by Parker (ze/zir) at all medical visits. Terry also has a strong network of friends, some of whom you have occasionally seen in the waiting room with Parker following appointments. Parker is listed as Terry’s emergency contact. Terry values spending time in nature when possible and is an avid reader.
Disease and Social History. You first met Terry nine months ago when she was diagnosed with Stage II HER2-positive breast cancer. Terry has progressed through multiple lines of treatment. Her disease has spread to her lumbar spine (L4–L5) and sacrum and she is now reporting constant moderate to severe pain in her lower back that is affecting her ability to exercise or walk for extended amounts of time. The pain worsens with movement and is no longer responding to opioids. Terry appears more anxious than normal and is now expressing significant worry about “what comes next”. An office practice nurse (OPN) on your team shared with you that Terry has recently expressed a strong fear of dying before she is “ready”. The OPN also mentions that Parker has been tearful on several recent phone calls and is concerned about how ze will take care of Terry.
Task. Today Terry and Parker are coming in to discuss next steps. Your task is to elicit their needs and goals using the strategies, skills, and process tasks from the *Inclusive Cancer Care for LGBTQ+ Patients’ Families* blueprint.
**Case 2**
Character History. Jennie Barton (she/them) is a physical therapist who works with professional athletes in a private practice. Jennie is originally from the Midwest and has been in New York City for about three years. Jennie is a transgender woman post chest (“top”) gender affirmation surgery and is in a relationship with Marc (he/him). Her mother Britton (she/her) is also very involved in Jennie’s care. Jennie and Marc have been living together for about two years and make life and health decisions together with Britton’s input.
Disease and Social History. Jennie presents today after several weeks of intermittent abdominal pain and night sweats accompanied by frequent bouts of nausea and diarrhea. Her appetite has decreased substantially over the past month and she has lost about 10 lbs in total. Jennie’s abdominal computed tomography (CT) scan shows an 8 × 4.5 cm peritoneal mass and the biopsy is positive for adenocarcinoma. Jennie is here to discuss possible treatment options, including first-line chemotherapy and surgical options. However, she appears to be in significant emotional distress in addition to her ongoing reports of pain, which have become more severe over the past few days. She is accompanied by Marc, who also appears upset.
Task. Today, Jennie and Marc are coming in to discuss treatment options. Your task is to provide empathic communication using the strategies, skills, and process tasks from the *Inclusive Cancer Care for LGBTQ+ Patients’ Families* blueprint.

**Table 2:** Key takeaways with exemplar focus group data.

Theme	Focus Group Data
Key takeaways	I liked how you broke the [exemplar videos] down into different skills… showing the videos of that is so importantly for people who just can’t wrap their heads around [LGBTQ+ inclusive care]. (P2) Learning how to do the introduction—asking for the pronoun—that is something I typically never do. I sort of just assume… that’s something I’m going to take away from this. (P7)
	The [didactic] did a fantastic job of contextualizing everything—it was a necessary and very useful part of this whole run of show and, in particular, the slides that read ‘what discrimination might look like’ was particularly germane and important to see. (P9) The idea of… having the person define their family… Because the whole idea of biological family and chosen family—that I imagine could be a really important skill set for this particular population. (P9) I think the content flowed well, the cases worked really well, I’m incredibly impressed and excited by it. I’m blown away by the quality. (P10) The first word that comes to mind is ‘grace’—this is next level—not only are we dealing with setting the agenda and the whole medical issue but now this awareness, and how programmed cisgender normative things are within me [even as a member of the LGBTQ+ community]. (P11) I thought the [rationale for why we are doing this training provided in the didactic] was really effective, I especially appreciated the statistics around the percentage of adults who identify—I was very surprised the… magnitude of people who identify as bisexual… I think the piece that could be amplified… is if we could have more statistics and more meat on the bones on [this population with serious illness to bridge the gap between the national level data] and who you might expect to see in your clinic… something that makes it a little more immediate. (P12) One thing that stuck out to me was when the clinician used the exact language that the patient used that was related to their sexual [orientation] or gender identity… in our role play [for the patient who had] top surgery… saying like, “…You just went through top surgery” versus, “Oh, you just had ‘[gender reassignment]’ surgery”…. Using the [patient’s] language could be something that we can incorporate into empathic communication. Or… [the patient introduces] someone as [their] husband, wife, and then the clinician saying, “partner”—like you being empathic by using the precise language used by the patient. That’s one thing [I will take away]. (P12) Everything was well paced, engaging, and you did a great job differentiating the content so all learners could be engaged… [The] small groups were great. People get nervous and don’t want to mess up, it is easier to be vulnerable in small groups. (P13) The presentation was really amazing in terms of the literature about the whole issue, the need for [the training], and for those of us who do clinical work, there’s so much need for communication around this and improving communication skills among clinicians… the actors were amazing… it was very helpful to work with these actors on this particular case and get feedback from them and be educated by them. (P14)

**Table 4:** Inclusive cancer care for LGBTQ+ patients’ families: communication blueprint.

Strategy	Skills	Process Tasks
Make introductions	Ask open questions (name, gender, sex, pronouns) Check patient preferences (regarding family and primary caregiver roles) Clarify relationships between patient and others present	Introduce yourself using pronouns Round of introductions and orientation to relationships Verbalize that the environment is intended to be safe and respectful Avoid being heteronormative and cis-normative (e.g., avoid assumptions)
2. Agenda Setting	Declare agenda items Invite agenda items Negotiate agenda (if appropriate)	Sit at eye-level Express willingness to help Actively include all individuals per patient guidance regardless of legal or biological relationship
3. Respond empathically to all family members	Encourage expression of feelings Acknowledge (potential differences between viewpoints of family members) Validate Normalize anxiety proportional to intensity Praise efforts	Maintain eye contact (if appropriate) Allow time to integrate; use silence Identify any members considered to be “at risk” or a concern to others Avoid premature reassurance
4. Invite patient and family members to engage in discussion about patient care.	Ask open questions (about social support, family of choice and/or biological family, person accompanying the patient, non-involvement, etc.) Endorse question asking Check preferences (for involving others in medical decision-making)	Introduce joint decision-making Explore the relational dynamics between the patient and other family members present
5. Check patient’s and family’s understanding of the illness and prognosis.	Ask open questions; make open-ended statements Check understanding (illness, concerns, etc.) Acknowledge Validate	Recognize both concordance and divergence of family members’ views Respect and include culturally sensitive views Honor protective urges and expressed desire to help Make partnership statements
6. Identify family’s concerns about their management of key symptoms or care needs	Ask open questions; make open-ended statements Check understanding Clarify Summarize	Use circular questioning to identify medication or treatment concerns Assess concerns about (a) extra help or support needed; (b) financial issues; (c) sense of helplessness; (d) fears/worries about mistreatment or discrimination Co-identify coping strategies and solutions to problems
7. Identify family strengths and affirm commitment and mutual support for each other	Ask open questions; make open-ended statements Praise family efforts Acknowledge Validate	Review family traditions, spirituality, cultural norms Identify family strengths Affirm their level of commitment and mutual support Endorse resilience Connect to necessary resources Ask about healthcare agent, advance directives, and other decision-making discussions
8. Close the consultation.	Check understanding Invite questions Endorse question-asking Summarize Review next steps	Reinforce joint decision-making

## Data Availability

The de-identified focus group transcript data the informed the conclusions of this article are available from the authors upon request.
